# Restriction Site Extension PCR: A Novel Method for High-Throughput Characterization of Tagged DNA Fragments and Genome Walking

**DOI:** 10.1371/journal.pone.0010577

**Published:** 2010-05-11

**Authors:** Jiabing Ji, Janet Braam

**Affiliations:** Department of Biochemistry and Cell Biology, Rice University, Houston, Texas, United States of America; Cinvestav, Mexico

## Abstract

**Background:**

Insertion mutant isolation and characterization are extremely valuable for linking genes to physiological function. Once an insertion mutant phenotype is identified, the challenge is to isolate the responsible gene. Multiple strategies have been employed to isolate unknown genomic DNA that flanks mutagenic insertions, however, all these methods suffer from limitations due to inefficient ligation steps, inclusion of restriction sites within the target DNA, and non-specific product generation. These limitations become close to insurmountable when the goal is to identify insertion sites in a high throughput manner.

**Methodology/Principal Findings:**

We designed a novel strategy called Restriction Site Extension PCR (RSE-PCR) to efficiently conduct large-scale isolation of unknown genomic DNA fragments linked to DNA insertions. The strategy is a modified adaptor-mediated PCR without ligation. An adapter, with complementarity to the 3′ overhang of the endonuclease (*Kpn*I, *Nsi*I, *Pst*I, or *Sac*I) restricted DNA fragments, extends the 3′ end of the DNA fragments in the first cycle of the primary RSE-PCR. During subsequent PCR cycles and a second semi-nested PCR (secondary RSE-PCR), touchdown and two-step PCR are combined to increase the amplification specificity of target fragments. The efficiency and specificity was demonstrated in our characterization of 37 *tex* mutants of *Arabidopsis*. All the steps of RSE-PCR can be executed in a 96 well PCR plate. Finally, RSE-PCR serves as a successful alternative to Genome Walker as demonstrated by gene isolation from maize, a plant with a more complex genome than *Arabidopsis*.

**Conclusions/Significance:**

RSE-PCR has high potential application in identifying tagged (T-DNA or transposon) sequence or walking from known DNA toward unknown regions in large-genome plants, with likely application in other organisms as well.

## Introduction

Linking gene identity to function is critical for genetic approaches to unravelling complex biological phenomena. For mutant genes disrupted by DNA insertions, the DNA insertion acts as a tag to enable the identification of the mutated gene. Obtaining flanking DNA is also valuable for isolating sequences upstream or downstream from a gene fragment. Unfortunately, especially for high-throughput screens, the current methods of isolating flanking DNA sequence, are less than ideal. There are three types of PCR-based techniques for walking in an unknown region from a known genomic fragment. The first type including TAIL-PCR uses nested specific primers from the ends of known region and degenerate primers that anneal randomly with the genome to obtain unknown flanking fragments [1], [2]. The second one usually digests the genomic DNA with a restriction enzyme to generate an overhang followed by ligation of a complementary adaptor. The primers derived from the adaptor and known sequence amplify the flanking sequences through successive rounds of PCR [3]–[12]. The third type such as inverse PCR (iPCR) begins with the digestion of genomic DNA with a restriction enzyme like the second one, however, subsequent intramolecular ligation generates a small DNA circle. Two primers designed in opposite direction from the known fragment could amplify the unknown junction region [13]–[18]. TAIL-PCR and iPCR have been used widely for identifying genes from *Arabidopsis* and rice. TAIL-PCR usually requires 3 rounds of amplification and special treatment of PCR samples before direct sequencing, and non-specific products are often a problem. iPCR requires sufficiently long sequences for two pairs of nested primers and presence of two appropriate restriction sites within an amplification range. Adaptor-based PCR usually suffers from non-specific amplification from the adaptor primers, and panhandle suppression may be inadequate especially when genome in question is very complex.

We have modified adaptor-based PCR such that non-specific amplification is reduced and ligation is avoided. Specifically we designed a novel PCR strategy called Restriction Site Extension PCR (RSE-PCR). Genomic DNA targets are specifically and efficiently amplified through two rounds of PCR. During the first cycle of the first round RSE-PCR (Primary RSE-PCR), a short extension of 5 seconds extends the 3′ end of the endonuclease restricted DNA fragments through a 5 bp terminal complementary to the 3′end of the 1^st^ adaptor primer. Simultaneously, this 1^st^ adaptor primer cannot complete its extension in such a short period along the majority of genomic DNA templates in the range of kilobases long. During the subsequent cycles and the second round of semi-nested RSE-PCR (Secondary RSE-PCR), touchdown and two-step PCR are combined to further enhance the amplification specificity.

The success of this novel strategy is demonstrated in the isolation of T-DNA flanking sequences from 23 out of 37 *Arabidopsis* mutants of interest, and unknown fragment for a particular gene of maize. The ease and specificity of RSE-PCR prove the efficacy of this approach toward high throughput application in genetics and genome walking in diverse organisms, including those of large complex genomes.

## Materials and Methods

An ethics statement is not required for this work.

### Genomic DNA isolation and restriction

Genomic DNA was isolated from young leaves of *Arabidopsis* and maize as described [19]. One µL (500 ng to 1 µg) of genomic DNA was digested with 10 units of restriction endonuclease generating 3′ overhangs in a 100 µL volume containing 1×BSA,1×buffer and 1 µl RNase A(10 µg/µL) for 3 hours under appropriate temperatures. The restriction endonucleases were subsequently heat inactivated.

### PCR primers and conditions

All primers were synthesized by GenoMechanix (Gainesville, FL) or Invitrogen, and are summarized in Table 1. One microlitre of the above restricted genomic DNA was added to a 10 µL primary RSE-PCR reaction comprising 0.5 µL of each primer (10 µM, one is JL270, BIL1 or LB1; the other is AdKpnI, AdNsiI, AdPstI, or AdSacI), 1×PCR buffer, 0.5 µl of 50 mM MgCl_2_, 0.5 µL of dNTP (2.5 mM each), and 0.25 U of Platinum Taq Polymerase (Invitrogen). The primary RSE-PCR program was performed as shown in Table 2. 190 µL of autoclaved ddH_2_O was added to each sample to make a 20 fold dilution after amplification, from which 1 µL was removed for the secondary RSE-PCR. The secondary RSE-PCR contained the same ratio of reagents in a 20 µL volume except with the nested specific primer, such as JL202, BIL2 or LB2 from known sequences, and the 2^nd^ general adaptor primer (AP), as described in Table 2.

**Table 1:** The oligonucleotides used in this study.

Name	Primer sequence	Primer use
AdKpnI	GTAATACGACTCACTATAGGGCGTACC	1^st^ Adaptor primer
AdNsiI	GTAATACGACTCACTATAGGGCTGCAT	1st Adaptor primer
AdPstI	GTAATACGACTCACTATAGGGCTGCAG	1st Adaptor primer
AdSacI	GTAATACGACTCACTATAGGGCAGCTC	1st Adaptor primer
AP	GTAATACGACTCACTATAGGGC	2^nd^ general adaptor primer
JL270	TTTCTCCATATTGACCATCATACTCATTG	1st PCR primer from pSuperTag2
JL202	CATTTTATAATAACGCTGCGGACATCTAC	2^nd^ PCR primer from pSuperTag2
BIL1	TCCTTTCGCTTTCTTCCCTTCCTTTCTC	1st PCR primer for SALK lines
BIL2	GGGCTCCCTTTAGGGTTCCGATTTAGTG	2nd PCR primer for SALK lines
LB1	GCCTTTTCAGAAATGGATAAATAGCCTTGCTTCC	1st PCR primer for SAIL lines
LB2	GCTTCCTATTATATCTTCCCAAATTACCAATACA	2^nd^ PCR primer for SAIL lines
ZmPFR13	CGTGAGACAAATTGGTGCACATCAAAAC	1^st^ primer of ns2 for 5′ genome walker
ZmPFR14	CAGAGAGCTTCGCAGTGATCCTTTTTGA	2^nd^ primer of ns2 for 5′ genome walker

**Table 2:** Cycling parameters for RSE-PCR.

PCR reaction	Cycle number	Thermal condition	Note
Primary	1	94°C, 2 min	
	1	94°C, 10 sec; 45°C, 20 sec; 72°C, 5 sec	3′ restriction site extension along the short template of the 1^st^ adaptor primer
	11	94°C, 10 sec; 72°C (temperature decreased 1°C/cycle until 62°C), 3 min	touchdown and two-step PCR
	19	94°C, 10 sec; 67°C, 3 min	two-step PCR
	1	67°C, 8 min	
Secondary	1	94°C, 2 min	
	11	94°C, 10 sec; 72°C (temperature decreased 1°C/cycle until 62°C), 3 min	touchdown and two-step PCR
	19–29	94°C, 10 sec; 67°C, 3 min	two-step PCR
	1	67°C, 8 min	

### Gel analysis and DNA sequencing

5 µL of the secondary RSE-PCR products were loaded in 1.2% agarose gel stained with ethidium bromide in a 1×TAE or 1×TBE buffer and visualized under a UV illumination system. The remaining 15 µL PCR products were purified through Sephadex G-50 column and subject to sequencing (Lone Star Labs, Houston, TX).

## Results and Discussion

### 1. Principle of RSE-PCR and optimization of PCR parameters

In 1993, Upcroft and Healey employed PCR priming from the *Sac*I restricted *Giardia duodenalis* (an intestinal protozoan parasite, genome size = ∼12 Mb) to successfully extend the 5′ flanking fragment of a drug resistance related gene [20]. Although there was no description of their PCR procedure, their idea could be extended and tested in large scale plant genetics. We designed the 1^st^ adaptor primers containing a core part of 22 bp (GTAATACGACTCACTATAGGGC, a derivative from Genome Walker upper adaptor strand (Clontech) and a 3′ terminus of 5 bp (GTACC for *Kpn*I, TGCAT for *Nsi*I, TGCAG for *Pst*I, and AGCTC for *Sac*I) as shown in Table 1. Theoretically, the probability for a restriction site of a six base pair endonuclease is 1 out of every 4^6^ (4096) base pairs, meaning that the average size of the restricted genomic DNA is about 4 Kb. If the sequence around the middle of a fragment is known, the isolation of its flanking 5′ and 3′ parts (about 2 Kb each) will be compatible with the amplification capacity of Platinum Taq Polymerase (Invitogen). The chance of successful isolation will be further increased through separate digestions with four different endonucleases.

**Figure 1:**
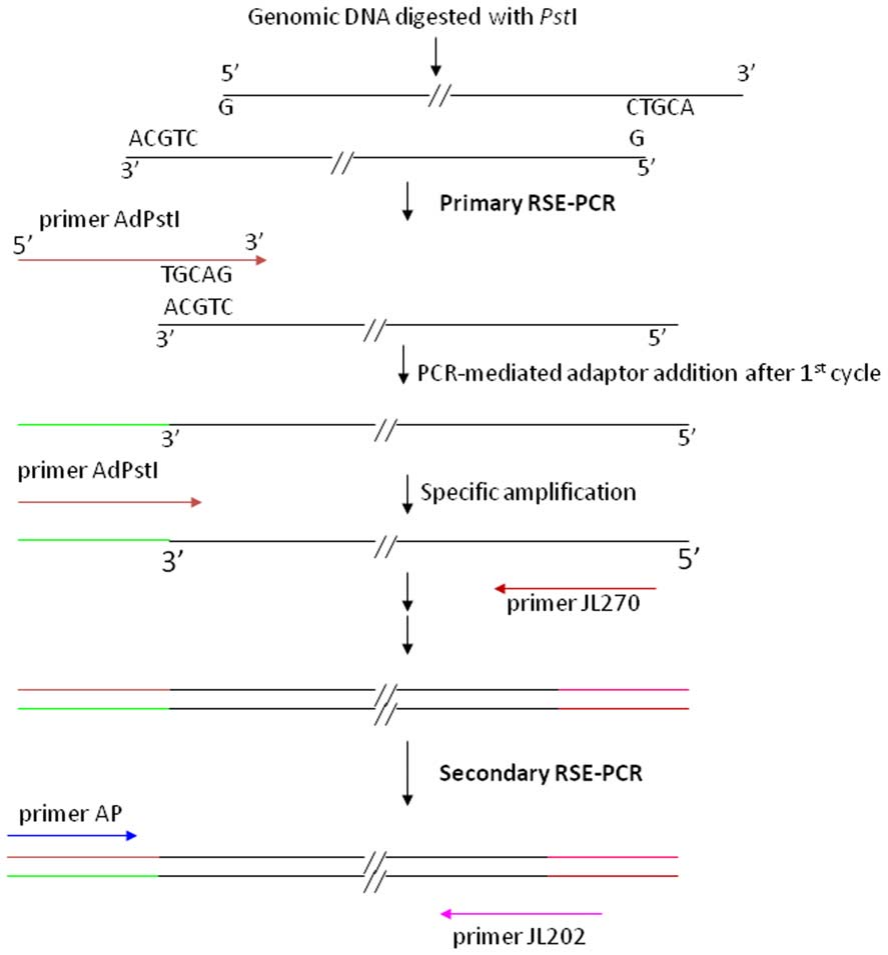
A general scheme for RSE-PCR. Each DNA sample is digested separately with four different endonucleases (*Kpn*I, *Nsi*I, *Pst*I or *Sac*I). During the primary RSE-PCR, a short extension of the first cycle extends the 3′ end of the endonuclease (*Pst*I as an example) restricted DNA fragments through a 5 bp terminal complementary to the 3′end of the 1^st^ adaptor primer (AdPstI), whereas the extension of the 1^st^ adaptor primer (AdPstI) along the majority of genomic DNA templates is not completed (not shown here). Subsequent specific exponential amplification of the target is favored through the combination of touchdown, two-step and semi-nested PCR strategy (secondary RSE-PCR), and driven by T-DNA (for example, JL270 from pSuperTag2 for primary RSE-PCR and JL202 from pSuperTag2 for secondary RSE-PCR) or gene specific primer. In this case, the 5′ flanking sequence from a known fragment will be extended. The 3′ flanking sequence will be isolated if nested reverse primers are used from the known fragment. Note the size of primers and genomic DNA fragment are not to scale.

During the primary RSE-PCR, a 5-second extension during the first cycle extends the 3′ end of the endonuclease restricted DNA strands through a 5 bp terminal complementary to the 3′end of the 1^st^ adaptor primer, whereas the extension of the 1^st^ adaptor primer along the majority of genomic DNA templates is not completed. Subsequent specific exponential amplification of the target is favored through the combination of touchdown, two-step and semi-nested PCR strategy and driven by primers from a known fragment such as T-DNA border sequence. This will give rise to the 5′ flanking sequence of a known fragment (Figure 1). However if nested reverse primers are used, the 3′ flanking sequence could be isolated from a known sequence. Five microlitres of the secondary PCR products are gel-checked as detailed in Materials and Methods, and if the result is positive, the remaining 15 µL PCR products are purified through Sephadex G-50 and subject to sequencing.

### 2. Isolating T-DNA flanking sequence in *Arabidopsis* transformed with different vectors

**Figure 2:**
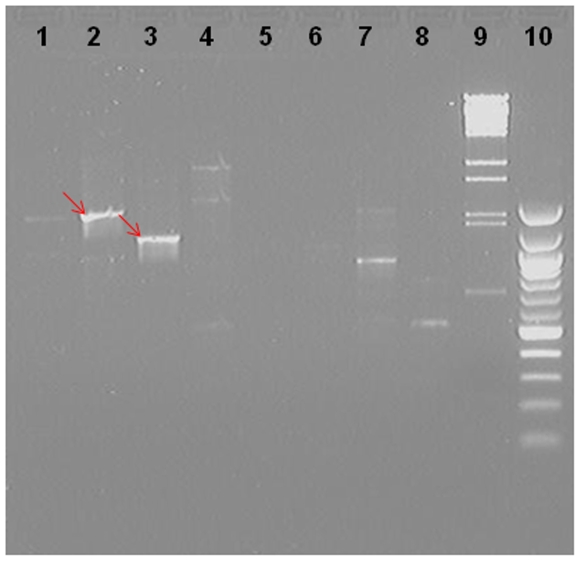
Gel image of one representative *tex* mutant (*tex34*) after two rounds of RSE-PCR. Lanes 1–4 were *tex34* restricted with *Kpn*I, *Nsi*I, *Pst*I and *Sac*I, while lanes 5–8 were *tex34* without any digestion. Primers AdKpnI (lanes 1 and 5), AdNsiI (lanes 2 and 6), AdPstI (lanes 3 and 7) and AdSacI (lanes 4 and 8) were used respectively in four primary RSE-PCRs together with JL270 from pSuperTag2, while primers JL202 from pSuperTag2 and AP were for all four secondary RSE-PCRs. Lanes 9 and 10 were 100 bp and lamda BstEII ladders. Five µl of each secondary RSE-PCR products were loaded in 1.2% agarose gel stained with ethidium bromide in a 1×TBE buffer. Note that the arrows represented the specific amplification.

To elucidate molecular mechanisms involved in the complex regulation of the *TCH4* (*TOUCH4*) gene [21], one transgenic line harboring the −258 to +48 of *TCH4* sequences fused to *LUC* in Col-0 background was mutagenized with pSuperTag2 vector to generate T-DNA insertion mutations [22], [23]. Genetic screens identified 37 mutants, which showed altered *TCH4* expression (*tex*) after heat shock. Previous attempts with TAIL-PCR worked with only one mutant out of 37 *tex* mutants (unpublished data, Luis & Braam). Using RSE-PCR, sequences flanking T-DNA insertions were isolated and sequenced from 23 out of 37 *tex* mutants (Table 3). Figure 2 shows the representative RSE-PCR products from one *tex* mutant digested with four endoenzymes. The RSE-PCR product size ranged from about 300 bp to nearly 3 Kb (data not shown). All the purified RSE-PCR products sequenced with JL202 primer contained T-DNA left border sequence and genomic sequence from *Arabidopsis*. Flanking sequences in the remaining 14 *tex* mutants failed to be isolated possibly due to tandem insertion, lack of intact T-DNA border sequence, DNA rearrangement, or complicated DNA context [24], [25]. One *tex* mutant contains two insertions. 6 *tex* mutants contain insertions in exons or introns, 4 downstream of protein coding regions and 14 upstream of protein coding regions.

**Table 3:** T-DNA insertion sites in 23 *tex* mutants obtained with RSE-PCR.

*tex*	Insertion
31	367bp of 5′end of AT4G02600 (similar to seven transmembrane MLO family protein/MLO-like protein 15 (MLO15)
32	340bp of 5′ end of AT4G02590
34	555bp of 5′end of bHLH family protein (AT4g02590)
38	exon of AT4G15050(weak similarity to phosphoglucose isomerase B(pgiB), Mycoplasma genitalium, endomembrane system locaton)
39	739bp upstream of CD of AT1G19650 (SEC14 cytosolic factor, putative/phosphoglyceride transfer protein)
40	3479 bp of 5′ end of AT5G28430
43	4130 bp upstream of U3 snRNA (AT5G53902)
45	3′UTR (94bp downstream of CD) of AT3G01630 (similar to nodulin-related)
46	1358bp upstream of CD of AT4g26170 (putative transcription factor)
49	exon of AT4g19900 (glycosyl transferase-related)
50	57bp of 5′end of AT3G57400 (N-terminal protein myristoylation)
51	399 bp of 5′ end of AT5G23850 (Lipopolysaccharide-modifying protein:IPR006598)
54	40 bp of 3′ end of AT3G13410
55	exon of AT5G53390 (FOP mRNA for FOLDED PETALS)
85	9 Kb upstream of 5′UTR of AT1G66150 (TRANSMEMBRANE KINASE 1)
87	2982bp of 5′end of AT5G67390
87	562 bp downstream of CD of At2g16290 (F-box family protein)
88	633 bp of 3′ end of AT4G36030 (armadillo/beta-catenin repeat family protein)
89	4835bp of upstream of CD of AT5G50910 (located in endomembrane sysytem)
91	7221 bp of 5′end of homeodomain transcription factor-like (AT5G66700)
92	first intron of AT5G46820 (contains similarity to carboxyl-terminal proteinase)
93	AT3G11100 disrupt expressed protein, similar to 6b-interacting protein 1 (NtSIP1) (Nicotiana tabacum)
95	965bp of 5 end of At2g05980 (putative non-LTR retroelement reverse transcriptase)
97	exon insertion (AT1G64790) translational activator family protein, similar to HsGCN1 (Homo sapiens)

In addition, we found that RSE-PCR also works with other vectors commonly used in SALK and SAIL T-DNA insertion lines. *xth22-A* (SAIL_158_A07) and *xth24-1* (SALK_005941.51.20.x) mutants insertion sites were successfully analyzed; the nested primers from the left borders of the vectors used in generating SAIL (LB1 and LB2) and SALK (BIL1 and BIL2) lines are listed in Table 1.

### 3. Isolation of multiple insertions in a single line

Multiple bands could be amplified after the secondary RSE-PCR, which suggests the presence of several T-DNA insertions in the line. After the gel separation of the bands, pipette tips of 1–200 µl were used to pick up a tiny piece of agarose gel directly from individual PCR bands under UV light. These were resuspended in 20 µL of autoclaved water by pippetting up and down several times. Then one microlitre was used for another round of PCR with the same primers and cycling program as in the secondary RSE-PCR. The PCR product was gel checked and purified as described above and subject to sequencing. As an example, *tex87* mutants were found to contain two T-DNA insertions: one is 2982bp of 5′end of AT5G67390, and the other 562 bp downstream of At2g16290 (F-box family protein).

### 4. Isolating unknown sequence from a particular known gene sequence in different plant species

The above work suggests that as long as the sequence of a DNA fragment is known, the specific flanking sequence can be isolated; therefore, we next tested the feasibility of the approach in more complex plant genomes. Maize *ns2* gene from B73 inbred line was specifically amplified after *Sac*I restriction (AdSacI and ZmPFR13 for the primary RSE-PCR, and AP and ZmPFR14 for the secondary RSE-PCR). Sequencing with primer ZmPFR14 recovered 863 bp of readout, which was the same obtained previously with Genome Walker Kit from Clontech [26]. This data suggests that RSE-PCR can substitute for genome walker kit for gene cloning.

Together, the data here indicate that a new strategy, RSE-PCR, has high potential application in identifying tagged (T-DNA or transposon) sequencing or walking from known DNA toward unknown regions in large-genome plants, with likely application in other organisms as well.
